# Computational simulation of the bone remodeling using the finite element method: an elastic-damage theory for small displacements

**DOI:** 10.1186/1742-4682-10-32

**Published:** 2013-05-13

**Authors:** Ahmed Idhammad, Abdelmounaïm Abdali, Noureddine Alaa

**Affiliations:** 1 Laboratory of Applied Mathematics and Computer Science (LAMAI), Faculty of Sciences and Technics, Cadi Ayyad University, Abdelkrim El Khattabi Avenue, Marrakech, Morocco

**Keywords:** Bone remodeling, Damage, Elasticity, Small displacements hypothesis, Bone density, Femur, Finite element, Variational formulation, Biomechanics, Computation simulation

## Abstract

**Background:**

The resistance of the bone against damage by repairing itself and adapting to environmental conditions is its most important property. These adaptive changes are regulated by physiological process commonly called the bone remodeling. Better understanding this process requires that we apply the theory of elastic-damage under the hypothesis of small displacements to a bone structure and see its mechanical behavior.

**Results:**

The purpose of the present study is to simulate a two dimensional model of a proximal femur by taking into consideration elastic-damage and mechanical stimulus. Here, we present a mathematical model based on a system of nonlinear ordinary differential equations and we develop the variational formulation for the mechanical problem. Then, we implement our mathematical model into the finite element method algorithm to investigate the effect of the damage.

**Conclusion:**

The results are consistent with the existing literature which shows that the bone stiffness drops in damaged bone structure under mechanical loading.

## Introduction

Bone is the main constituent of the skeletal system enable to maintain substantially the shape of the body; to protect the internal organs; to store minerals and lipids; to participate in blood cell production; and to assist body movements by transmitting the force of muscular contraction from one part to another [1].

As a living tissue, bone is able to optimize its structure by redistributing its density under the influence of external forces. Since this publication of Wolff, many theories describing the redistributing of the bone density have been proposed [2-4].

This process, called bone remodeling, was formally developed later by Huiskes et al. using the concept that bone remodeling is induced by a local mechanical signal which activate the regulating cells and cause local bone adaptations [5,6].

However, when external forces are above a critical level, bone may become more susceptible to fracture by increasing the damage formation which is normally repaired [7,8].

Therefore, better understanding of bone remodeling process helps to prevent fractures and other kinds of diseases. Several works have been made to relate bone remodeling process to a mathematical point of view, and thereafter perform some computational simulations [6,7].

The overall aim of this study is to numerically simulate the proximal femur using an elastic-damage theory for small displacements. First, we describe the mechanical problem and we derive its variational formulation. Next, we propose a bone remodeling algorithm and we solve a two-dimensional femur problem by using the finite element method.

Finally, some two-dimensional computational simulations are presented, and the results are in clear agreement with those reported in literature.

## Background

### Geometry and material properties

**Figure 1:**
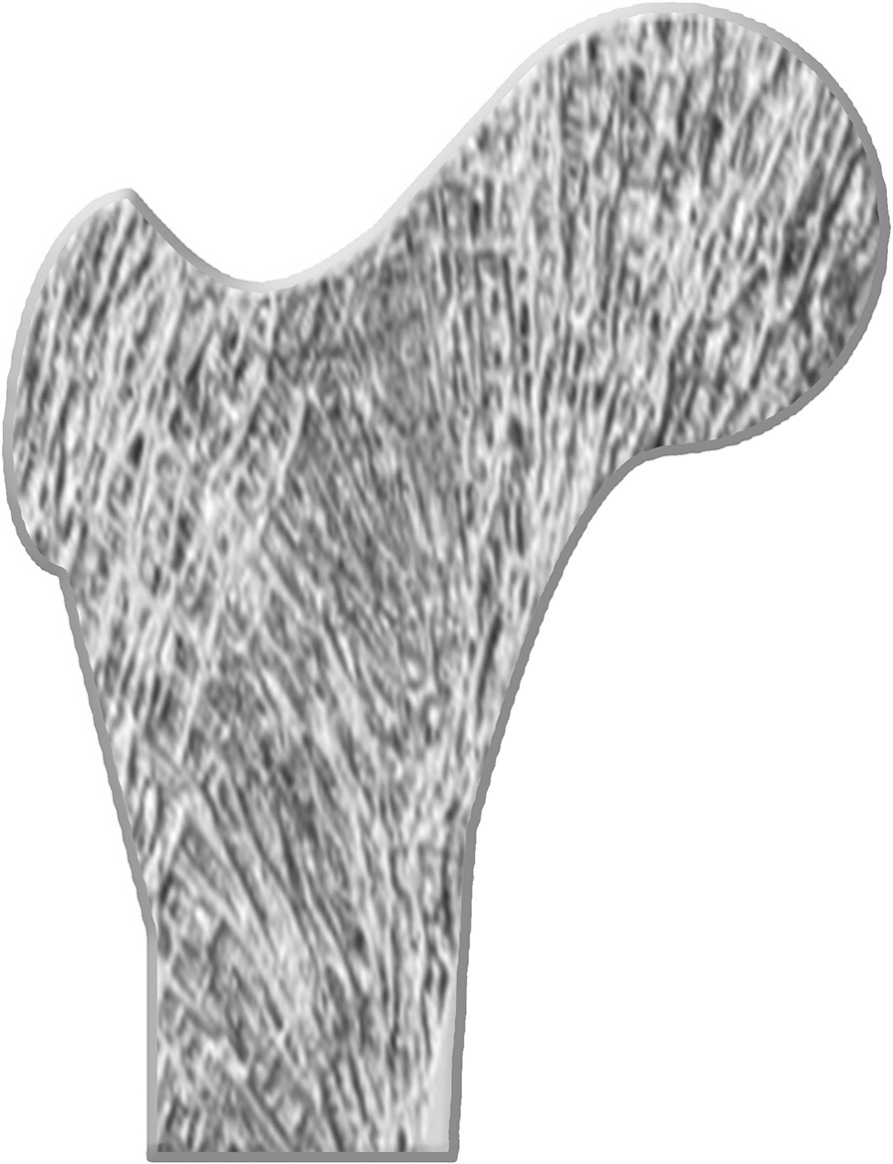
Model of a proximal femur.

We consider a two-dimensional model of a proximal femur as previous studies from the literature [9-11]. The suggested geometry is schematically shown in Figure 1.

Material properties of the femur are assumed as linear elastic, damageable, isotropic and homogeneous. The Poisson ratio is assigned to *υ* = 0.3 as referred from literatures [12,13]; the Young modulus (E) is computed from the bone density ρ using expression:

$$E=M{.\rho }^{\gamma }$$

where M and γ are positive constants.

### Elastic-damaged bone remodeling theory

The femur can be remodeled in response to external loads to give greater bone matrix in regions that are subjected to higher levels of stress. These external loads induce changes in the mechanical fields and damage to the femur, such as macro cracks or micro cracks [14,15].

This concept of damage was developed in the 1990s by the scientific community and particularly by Kachanov [16]. The elastic-damage model used in this contribution was initially developed by the French school and notably that of Jean Lemaitre [17,18]. It describes the constitutive behavior of the material by introducing a scalar variable D which quantifies the influence of microcracking.

In this theory of elastic-damage mechanics, the elastic modulus of the element may degrade gradually as damage progresses. Under the hypothesis of small displacements, the elastic modulus of damaged material is defined as follows:

$$\tilde{E}=\left(1-D\right).E$$

Where:

D is the degree of damage with 0 ≤ D ≤ 1

E is Young’s modulus of undamaged elasticity

$\tilde{E}$is the actual modulus of damaged elasticity. The damage is then expressed as the loss of stiffness.

We apply the theory of elastic-damage mechanics to bone remodeling process, and we use the previous equations to get:

$$\tilde{E}=M.{\tilde{\rho }}^{\gamma }$$

Where $\;\tilde{\rho }$ is the bone density that takes into account the damage as proposed by Abdali [19], which can be expressed by:

$$\;\tilde{\rho }=\rho \;{\left(1-D\right)}^{\frac{1}{\gamma }}$$

We assume that this function is bounded as:

$$\rho_{\min}\;\leq \;\tilde{\rho }\;\leq \;\rho_{\max}$$

Where:

ρ_min_ the minimal density corresponding to the reabsorbed bone

ρ_max_ the maximal density of cortical bone

Moreover, let ρ_0_ denote the initial bone density

Then, we introduce the evolution law of the damage suggested by Martin [7,20] through the equation:

$$D=D_{0}.\;e^{f_{d}\;t}$$

Where:

D_0_ is the initial damage

t is the time

f_d_ is the fatigue life of the bone devoid of the remodeling

### Governing equations

Let $Ω\subset R^{2}$, be a nonempty open bounded domain in $R^{2}$ with a Lipschitz-continuous boundary Γ = ∂ Ω. The boundary is split into two disjoint parts Γ_1_ and Γ_2_ where Γ_1_ is a fixed part of the border on which the femur is fixed, and Γ_2_ is also a part of the border, on which the forces F (F1, F2) are applied.

Everywhere below we use $\;S^{2}\;$ to denote the space of second order symmetric tensors and denote by n the unit outer normal vector to Γ. To simplify, we consider that the body occupying the set $\bar{Ω}=Ω\;\cup \;\Gamma$ isn’t being acted upon by a volume force of density f. Subsequent to modeling, both loads and boundary conditions are defined.

**Figure 2:**
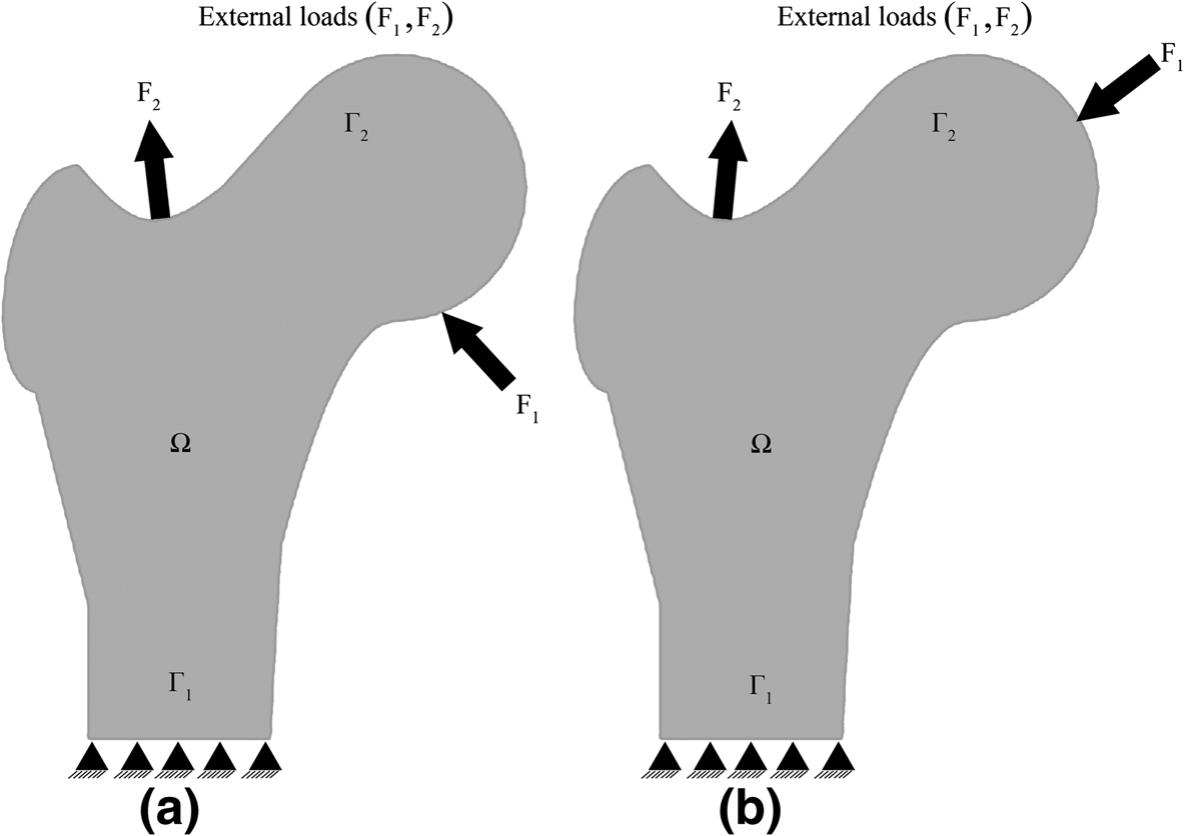
The different load locations, (a) load case 1, (b) load case 2.

Figure 2 shows the different load locations acting on the femur.

Finally, let $\;u:\;\bar{\Omega }\;x\;\;\left[0,T\right]\;\to R^{2}\;$ be the displacement field, $\sigma :\;\bar{\Omega }\;x\;\left[0,T\right]\;\to \;S^{2}$ the stress field, $\epsilon :\;\bar{\Omega }\;x\;\left[0,T\right]\;\to S^{2}$ strain tensor, $\;\tilde{\rho }:\;\bar{\Omega }\;x\;\left[0,T\right]\;\to \;\left[\rho_{\min},\;\rho_{\max}\right]$ the bone density function and T > 0 the time duration.

We recall that ϵ(u) is given by [9,21,22]:

$$\epsilon \left(u\right)=\frac{1}{2}\;\left(\left(\nabla u\right)+{\left(\nabla u\right)}^{T}\right)=\frac{1}{2}\;\left(\;\frac{\partial u_{i}}{\partial x_{j}}+\frac{\partial u_{j}}{\partial x_{i}}\right),\;i,j=1,2$$

Here ∇ stands for the gradient operator.

The constitutive law related the stress–strain relationship [9] is written as:

$$\sigma \left(u\right)=2.\mu \left(\tilde{\rho }\right).\epsilon \;\left(u\right)+\lambda \left(\tilde{\rho }\right).\mathrm{Div}\left(u\right).l$$

Where:

Div represents the divergence operator

l denotes the identity operator in $S^{2}\;$

$\mu \left(\tilde{\rho }\right),$$\lambda \left(\tilde{\rho }\right)$ are Lame’s coefficients, which are assumed to depend on the bone density denoted by $\tilde{\rho }.$

$\mu \left(\tilde{\rho }\right)$ is expressed as $\lambda \left(\tilde{\rho }\right)=\frac{E\left(\tilde{\rho }\right).\upsilon \left(\tilde{\rho }\right)}{\left(1+\upsilon \left(\tilde{\rho }\right).(1-2.\upsilon \left(\tilde{\rho }\right)\right)}$

$\lambda \left(\tilde{\rho }\right)$is expressed as $\mu \left(\tilde{\rho }\right)=\frac{E\left(\tilde{\rho }\right)}{2\left(1+\upsilon \left(\tilde{\rho }\right)\right)}$

with:

$E\left(\tilde{\rho }\right)$ Young’s modulus

$\upsilon \left(\tilde{\rho }\right)$ Poisson’s ratio

Many laws of bone remodeling have been published in the world mainly targeting the evolution of the bone density [5,6,20]. We adopt the law suggested by Huiskes et al. who used the strain energy density as the stimulus signal to control bone remodeling process [5,6,20,22].

The evolution of the bone density function [6,22] is obtained from the following nonlinear first-order ordinary differential equation:

$$\frac{\partial \rho }{\partial t}=\left\{\begin{array}{cc} B.\left(\frac{U\left(\sigma \left(u\right),\epsilon \left(u\right)\right)}{\rho }–\left(1+s\right)K\;\right) & \;\mathrm{if}\;\frac{U}{\rho }>\left(1+s\right)K\\ 0\;\mathrm{if} & \;\left(1-s\right)K\leq \frac{U}{\rho }\leq \left(1+s\right)K\;\mathrm{in}\;\bar{\Omega }\;x\;\left[0,T\right]\;\\ B.\left(\frac{U\left(\sigma \left(u\right),\epsilon \left(u\right)\right)}{\rho }-\left(1-s\right)K\;\right) & \;\mathrm{if}\;\frac{U}{\rho }<\left(1-s\right)K \end{array}\right)$$

where:

B, s and K are experimental constants

U(σ(u), ϵ(u)) is the strain energy density [9] that regulates the remodeling process given by:

$$U=\frac{1}{2}\;\sigma \left(u\right):\epsilon \left(u\right)$$

Let: be the double contraction of two tensors which yields a scalar.

Finally, equation system of the problem is defined as follows.

Problem: Find $\left(u,\tilde{\rho }\right)\;$ such that

$$\left\{\begin{array}{lll} -\mathrm{Div}\;(2.\mu \left(\tilde{\rho }\right).\epsilon \left(u\right)+\lambda \left(\tilde{\rho }\right).\mathrm{Div}\left(u\right).l=f & \mathrm{in}\;\Omega x\left[0,T\right] & \;\left(1\right)\\ \frac{\partial \tilde{\rho }}{\partial t}=F\left(\tilde{\rho },u\right) & \mathrm{in}\;\Omega x\left[0,T\right] & \;\left(2\right)\\ u=0 & \mathrm{on}\;\Gamma_{1}x\left[0,T\right] & \;\left(3\right)\\ \sigma n=F & \mathrm{on}\;\Gamma_{2}x\left[0,T\right] & \;\left(4\right) \end{array}\right)$$

Where

$\tilde{\rho }=\rho \;{\left(1-D_{0}.\;e^{f_{d}\;t}\;\right)}^{\frac{1}{\gamma }},$$\epsilon \left(u\right)=\frac{1}{2}\;\left(\left(\nabla u\right)+{\left(\nabla u\right)}^{T}\right),$$\lambda \left(\tilde{\rho }\right)=\frac{E\left(\tilde{\rho }\right).\upsilon \left(\tilde{\rho }\right)}{\left(1+\upsilon \left(\tilde{\rho }\right).(1-2.\upsilon \left(\tilde{\rho }\right)\right)},$

$\mu \left(\tilde{\rho }\right)=\frac{E\left(\tilde{\rho }\right)}{2\left(1+\upsilon \left(\tilde{\rho }\right)\right)},$$\sigma \left(u\right)=2.\mu \left(\tilde{\rho }\right).\epsilon \;\left(u\right)+\lambda \left(\tilde{\rho }\right).\mathrm{Div}\left(u\right).l,$

$$F\left(\tilde{\rho },u\right)=\left[B.\left(\frac{U}{\tilde{\rho }\;{\left(1-D_{0}.e^{f_{d}\;t}\right)}^{-\frac{1}{\gamma }}}–\left(1\pm s\right)K\right)–\frac{D_{0}f_{d}}{\gamma }.e^{f_{d}\;t}.{\left(1-D_{0}.e^{f_{d}\;t}\right)}^{-\frac{1}{\gamma }-1}.\tilde{\rho }\right].{\left(1-D_{0}.e^{f_{d}\;t}\right)}^{\frac{1}{\gamma }}\;$$

$$\;\frac{U}{\tilde{\rho }}>\left(1+s\right).K.{\left(1-D_{0}.e^{f_{d}\;t}\right)}^{-\frac{1}{\gamma }}\;\mathrm{or}\;\frac{U}{\tilde{\rho }}<\left(1-s\right).K.{\left(1-D_{0}.e^{f_{d}\;t}\right)}^{-\frac{1}{\gamma }}\;\mathrm{otherwise}=0$$

### Variational formulation

The variational formulation of this model consists in a variational equation for the displacement field.

The weak form of (eq 1, 3 and 4) reads as follows: we seek for the displacement field u ∈ V = {φ ∈ [H^1^(Ω)]^2^; φ = 0 on Γ_1_} such that

$$\underset{\Omega }{\int }2.\mu \left(\tilde{\rho }\right).\epsilon \;\left(u\right):\epsilon \;\left(v\right)+\lambda \left(\tilde{\rho }\right).\mathrm{Div}\left(u\right).\mathrm{Div}\left(v\right)\mathrm{dx}=\underset{\Omega }{\int }f.v\;\mathrm{dx}+\underset{\Gamma_{N}}{\int }F.v\;\mathrm{ds}\;\forall v\in V$$

Where v is the test functions.

### Time discretizations

The Runge–Kutta 2nd order method is a numerical technique used to solve the ordinary differential equation 2:

$${\tilde{\rho }}_{n+1}={\tilde{\rho }}_{n}+\Delta t.f\left(\;t+\frac{\Delta t}{2},\;{\tilde{\rho }}_{n}+\frac{\Delta t}{2}.f\left(t,{\tilde{\rho }}_{n}\right)\right)\;.$$

With:

$$f\left(t,{\tilde{\rho }}_{n}\right)=B.\left(\frac{U}{{\tilde{\rho }}_{n}}–\left(1\pm s\right)K\;\right)\;\frac{U}{{\tilde{\rho }}_{n}}>\left(1+s\right)K\;\mathrm{or}\;\frac{U}{{\tilde{\rho }}_{n}}<\left(1-s\right)K\;\mathrm{otherwise}=0\;$$

∆t is the time step size

${\tilde{\rho }}_{0}=\rho_{0}\;{\left(1-D_{0}\right)}^{\frac{1}{\gamma }}$ is the initial data

### The proposed algorithm

**Figure 3:**
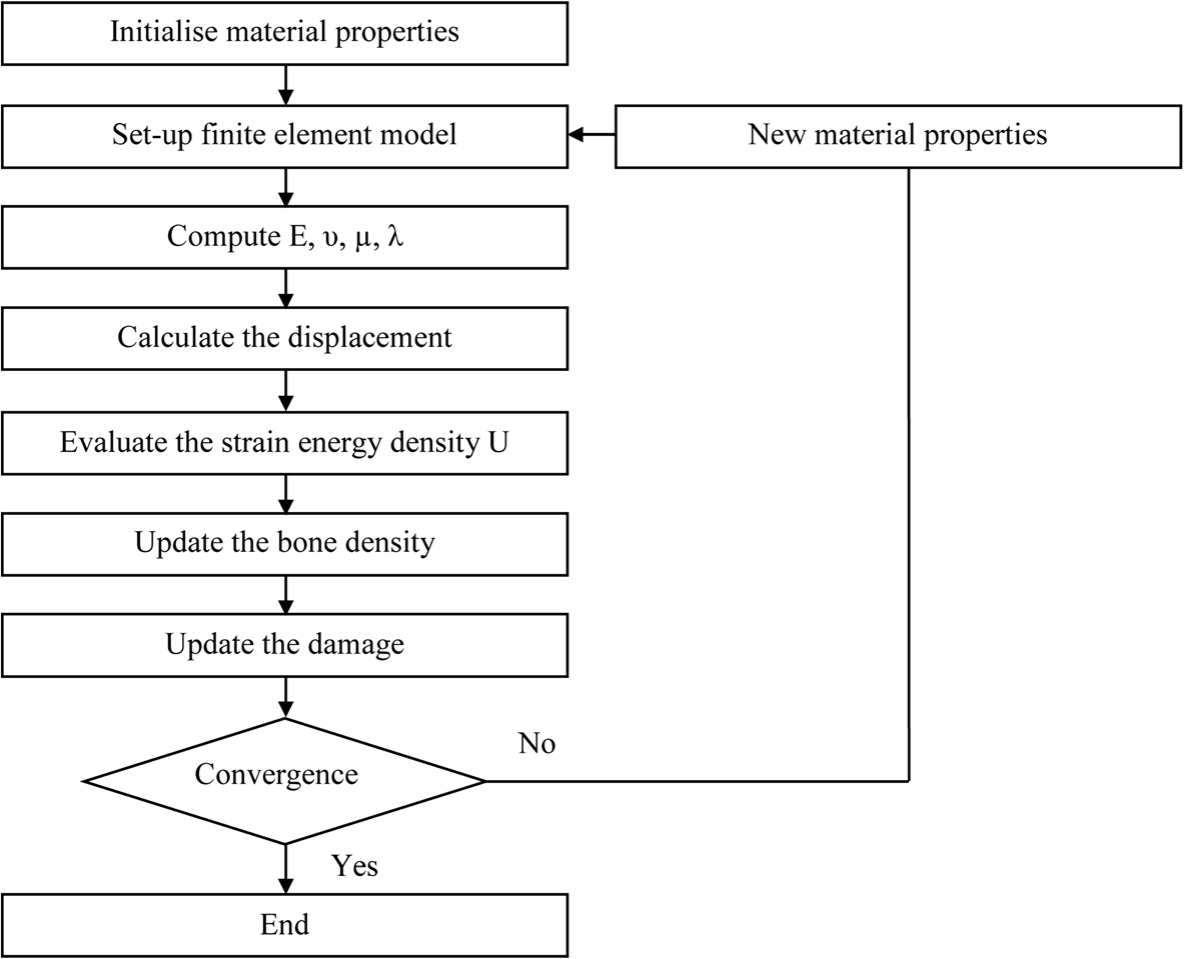
Schematic representation of the bone remodeling algorithm proposed.

Different algorithms are adopted to assign material properties into elements of the mesh model [23-25]. Some of these algorithms are based on the assumption without damage. The aim of this study is to simulate an elastic-damage of femur with finite element method. A schematic illustration of the hierarchical algorithm is presented in Figure 3.

The proposed algorithm can be summarized by the seven following steps:

Step 1. Define the global model: geometry, load conditions and initial bone density distribution. The remodeling is considered for initial model with a uniform density distribution of ρ_0_ = 0.8 g/cm^3^.

Step 2. Determine Young’s modulus, Poisson’s ratio and Lame’s coefficients.

Step 3. Calculate the displacement, by solving the linear variational equation of the displacement field.

Step 4. Evaluate the strain energy density U at each discrete location using the finite element method.

Step 5. Justify if the mechanical stimulus would cause bone apposition, bone resorption or equilibrium. Then, update the bone density.

Step 6. Update the damage.

Step 7. Check for convergence. The convergence criterion is imposed according to the change in mass during the iterative process. The final topology is obtained when the convergence criterion is satisfied; otherwise, the iterative process continues from Step 2.

## Results and discussion

As is well-known, it is of significance to explore the biomechanical behavior of bone. This work is aimed to simulate an elastic-damage femur in order to provide useful information on the geometrical topology and material properties of bone.

The following data [7,9,19,20,22,24] are employed:

ρ_min_ = 0.01 g/cm^3^, ρ_max_ = 1.74 g/cm^3^, K = 0.004 J/g, s = 0.1, ρ_0_ = 0.8 g/cm^3^, γ = 3, B = 1( g/cm^3^)^2^(MPa. UT)^- 1^, D_0_ = 0.8, f_d_ = 3 years, f = 0 N/m^2^, Δt = 10^- 5^UT, *υ* = 0.3, M = 3790 (Mpa)( g/cm^3^)^- 3^, (F_1_(case1) = 1000 N, F_2_(case1) = 1200 N), (F_1_(case2) = 1200 N, F_2_(case2) = 1500 N).

Several computational simulations are developed concerning bone remodeling of a proximal femur during mechanical stress, assuming the imposition of an elastic-damage in the domain. Also, we take a steady force and fixed constraint as the boundary condition of this model. The proposed algorithm described before is implemented in FreeFEM++ (see [26]), executing multiple simulations for different load cases. Two load cases are considered to evaluate their effect on the density distribution of bone.

**Figure 4:**
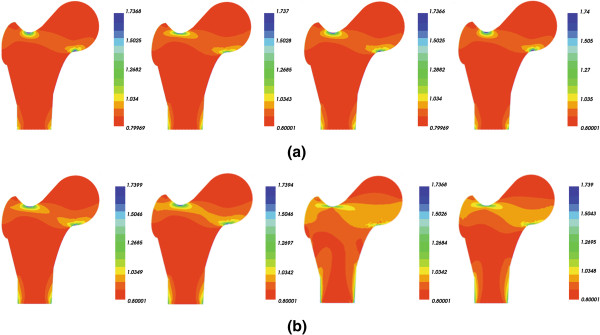
Bone densities from initial time (left) to final time (right) under load case 1, (a) undamaged femur, (b) damaged femur.

Figure 4 presents a comparison of the bone density distribution without and with damage in load case 1.

Shown in Figure 4(a) is the undamaged bone density distribution from initial time to final time. It can be seen that mechanical loading triggers the process of bone remodeling particularly in the area close to the load. In this area, the bone density is high compared to other areas.

Figure 4(b) plots the changes in the density distribution of damaged bone from initial time to final time. It results the increase in the rate of density during the initial time, and after that it gradually decreased. But it never reaches the density of an undamaged bone.

**Figure 5:**
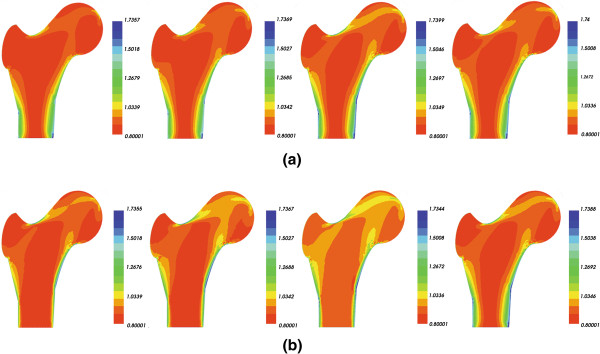
Bone densities from initial time (left) to final time (right) under load case 2, (a) undamaged femur, (b) damaged femur.

In Figure 5, the density distribution under load case 2 of an undamaged and damaged bone is shown. It is also possible to observe the same tendency as the previous figure.

The results of the study demonstrate that in the area close to the mechanical load, the bone density is higher than normal; and in the area remote from the mechanical load, the bone density is lower than other areas. The results are similar to those obtained by Li et al. [23], Sharma et al. [24] and Li et al. [27].

Comparing Figures 4 and 5, it is possible to observe that the decrease in bone density further leads to decrease in bone stiffness in terms of Young’s modulus, so to an increased risk of fracture. The same results were observed in the works of Tomaszewski et al. [28].

From this comparison, we showed that in a bone, the density decreases in a damaged structure at the initial time, then it can repair the damage itself to some extent at the final time. But this can only happen if the loading isn't so high that the self-repair mechanism can keep pace with the increasing damage [7,14,29,30].

The self-repair mechanism in this case is taken into account only the mechanical stimulus, although existing of many biological factors such as immunological, hormonal and haemodynamical stimulus; thus varying from one individual to another [24,27,31].

The work presented here may be applied to different models as well as to studies of orthopedic biomaterials and be helpful in further investigations.

## Conclusion

In the present study, a model simulating elastic-damage bone remodeling is presented by using a two-dimensional mathematical model and a numerical technique based on the finite element method. The effects of both strain and damage in bone structure have been examined.

The results presented in this paper show that in the solicited area, the bone density is important; and allowed us to observe a good agreement with literature findings.

Hence, from a biomechanical perspective it is better to simulate three dimensional femur bone by using the finite element method in order to obtain better understanding of the behavior of the bone.

## Competing interests

The authors declare that they have no competing interests.

## Authors’ contributions

All authors contributed to writing and improving the paper and approved the final manuscript.
